# CpG Oligonucleotides as Cancer Vaccine Adjuvants

**DOI:** 10.3390/vaccines3020390

**Published:** 2015-05-08

**Authors:** Hidekazu Shirota, Debra Tross, Dennis M. Klinman

**Affiliations:** 1 Department of Clinical Oncology, Tohoku University Hospital, Sendai 980-8577, Japan; E-Mail: Shirota@idac.tohoku.ac.jp; 2 Cancer and Inflammation Program, National Cancer Institute, Frederick, MD 21702, USA; E-Mail: Trossd@mail.nih.gov

**Keywords:** CpG, TLR, vaccine, cancer

## Abstract

Adjuvants improve host responsiveness to co-delivered vaccines through a variety of mechanisms. Agents that trigger cells expressing Toll-like receptors (TLR) activate an innate immune response that enhances the induction of vaccine-specific immunity. When administered in combination with vaccines designed to prevent or slow tumor growth, TLR agonists have significantly improved the generation of cytotoxic T lymphocytes. Unfortunately, vaccines containing TLR agonists have rarely been able to eliminate large established tumors when administered systemically. To improve efficacy, attention has focused on delivering TLR agonists intra-tumorally with the intent of altering the tumor microenvironment. Agonists targeting TLRs 7/8 or 9 can reduce the frequency of Tregs while causing immunosuppressive MDSC in the tumor bed to differentiate into tumoricidal macrophages thereby enhancing tumor elimination. This work reviews pre-clinical and clinical studies concerning the utility of TLR 7/8/9 agonists as adjuvants for tumor vaccines.

## 1. Introduction

Adjuvants are immunological agents that function to enhance the magnitude, breadth, quality and/or longevity of specific immune responses generated against co-administered antigens (Ag). Adjuvants are also used to reduce the dose and frequency of immunizations required to achieve protective immunity. Historically, vaccines were produced from live attenuated or heated inactivated organisms. While not appreciated at the time, those original vaccines contained bacterial contaminants that served as adjuvants [1]. 

There are several ways in which an adjuvant can promote immunity including:

(1) Stabilizing or entrapping the Ag to extend release and thus prolong immune stimulation;
(2) Promoting an inflammatory response at the site of Ag deposition thereby attracting activated macrophages and dendritic cells to improve Ag uptake and presentation;
(3) Presenting co-stimulatory signals to T and B cells to enhance induction of Ag-specific immunity.

There is considerable interest in identifying safer and more effective adjuvants to enhance the utility of novel vaccines targeting infectious pathogens, allergy and cancer. 

In support of these goals, immunologists and microbiologists have sought to elucidate the mechanism(s) of action of adjuvants. Notable success was achieved in the discovery of Toll like receptors (TLRs) and their role is promoting innate and adaptive immune responses, leading to a Nobel prize for Drs. Hoffmann and Beutler in 2011 [2,3].

## 2. Background Information Concerning TLRs

TLRs are an important component of the host’s pathogen sensing mechanism [4,5]. TLRs are typically classified into two families based on their localization: TLRs 1, 2, and 4–6 are expressed on the cell surface and sense bacterial cell wall components whereas TLRs 3 and 7–9 are expressed in endosomes and sense viral or bacterial nucleic acids [6]. The molecular structures recognized by TLRs have been evolutionarily conserved, are expressed by a wide variety of infectious microorganisms, and are termed pathogen-associated molecular patterns (PAMPs) [4,5]. The innate immune response elicited by TLR activation is characterized by the production of pro-inflammatory cytokines, chemokines, type I interferons and anti-microbial peptides. This innate response promotes and modulates the adaptive immune system. A common result is the expansion of Ag specific B cells that produce high affinity antibodies and of cytotoxic T cells including long-lasting memory cells that protect against subsequent infection through enhanced cytotoxic function targeting the effector phase [7,8].

Although several TLRs utilize similar signaling pathways, there are reproducible differences in the cytokine profile and adaptive immune response each elicits. Understanding which elements of the immune response are best supported by which TLR ligands should enable the development of adjuvants specifically tailored to enhance desired vaccination outcomes.

## 3. CpG ODN and TLR9

TLR9 recognizes and is activated by CpG motifs (consisting of a central unmethylated CG dinucleotide embedded within specific flanking regions) present at high frequency in bacterial DNA [9,10]. TLR9 molecules differ between species, with the structure of human *versus* mouse TLR9 varying by 24% [9]. There is also variation between species in terms of which cell types express TLR9. For example, the TLR9 receptor is present in rodent but not in primate macrophages and myeloid dendritic cells (DC). In humans, TLR9 is expressed primarily by plasmacytoid DC and B cells [11,12,13,14]. Reflecting their utility as vaccine adjuvants, B lymphocytes exposed to TLR9 agonists become more susceptible to activation by Ag [15,16,17] while TLR9 stimulated pDC produce type I interferons and more efficiently present Ag to T cells [18,19,20]. The signaling pathway triggered when CpG interacts with TLR9 proceeds through the recruitment of myeloid differentiation factor 88 (MyD88), IL-1R-associated kinase (IRAK), and tumor necrosis factor receptor-associated factor 6 (TRAF6) [5]. This signaling cascade subsequently leads to the activation of several mitogen-activated kinases (MAPK) and transcription factors (such as NF-kB and AP-1), culminating in the transcription of pro-inflammatory chemokines and cytokines [5].

In humans, four distinct classes of CpG ODN have been identified based on differences in structure and the nature of the immune response they induce. Although each class contains at least one motif composed of a central unmethylated CG dinucleotide plus flanking regions, they differ in structure and immunological activity. “K”-type ODNs (also referred to as “B” type) contain from one to five CpG motifs typically on a phosphorothioate backbone. This backbone enhances resistance to nuclease digestion and substantially prolongs *in vivo* half-life (30–60 min compared with 5–10 min for phosphodiester) [21]. K-type ODNs trigger pDC to differentiate and produce TNFα and stimulate B cells to proliferate and secrete IgM [22,23]. Unless otherwise mentioned, the studies described below reflect the activity of K ODN as these have been studied most extensively in pre-clinical and clinical trials.

“D”-type ODNs (also referred to as “A” type) have a phosphodiester core flanked by phosphorothioate terminal nucleotides. They carry a single CpG motif flanked by palindromic sequences that enables the formation of a stem-loop structure. D ODN also have poly G motifs at the 3' and 5' ends that facilitate concatamer formation. D-type ODNs trigger pDC to mature and secrete IFNα but have no effect on B cells [22,24]. C-type ODNs resemble K-type in being composed entirely of phosphorothioate nucleotides but resemble D-type in containing palindromic CpG motifs that can form stem loop structures or dimers. This class of ODN stimulates B cells to secrete IL-6 and pDC to produce IFNα [25,26]. P-Class CpG ODN contains double palindromes that can form hairpins at their GC-rich 3' ends as well as concatamerize due to the presence of the 5' palindromes. These highly ordered structures are credited with inducing the strongest type I IFN production of any class of CpG ODN [27,28].

## 4. Effect of CpG ODN on Human pDC and B Cells

In humans, TLR9 is expressed primarily by B cells and plasmacytoid DC (pDC) [29]. By comparison, multiple cells of the myeloid lineage including conventional DCs, monocytes and macrophages express TLR9 and respond to CpG ODN in mice [30]. pDC contribute to the initiation of many immune responses: they promote the generation of protective immunity to viral infection via their rapid and massive production of type I IFNs that support the generation of strong CTL responses [31,32,33]. Human pDC express TLRs 7 and 9 whereas myeloid DC (mDC) recognize TLRs 2, 3, 4, 5, 6 and 8 [29]. These divergent patterns of TLR expression support the hypothesis that distinct DC subsets generate unique/tailored responses optimized for the elimination of different pathogens [34,35]. Thus, CpG ODN should be particularly useful as adjuvants for vaccines targeting viral infections and cancer, both of which require the type of strong CTL response elicited by pDC activation [36,37]. 

TLR9 activation also induces human memory B cells to proliferate, undergo class switching to IgG2a and secrete antibodies in a T cell independent manner [38]. By comparison, naive human B cells express low levels of TLR9 and do not respond directly to CpG ODN [14]. Ag stimulation via the B cell receptor induces naive B cells to up-regulate TLR9 expression and acquire responsiveness to CpG DNA. The requirement that naive B cells interact with cognate Ag before acquiring responsiveness to CpG prevents polyclonal B cell activation and reduces the risk of autoimmunity [39]. This synergy between BCR ligation and CpG ODN stimulation was verified in studies using CpG-Ag complexes to enhance Ag-specific class switching *in vivo* and supports the use of CpG ODN as adjuvants for vaccines designed to induce strong humoral responses [39]. 

## 5. CpG ODN as Vaccine Adjuvants: Importance of CpG-Ag Co-Delivery

A number of preclinical (murine) studies examined the immunogenicity of CpG-adjuvanted vaccines. Most reported that CpG ODN enhanced both the humoral and cellular (Th1 cells and CTL) immune response elicited by vaccines against pathogens, allergens and/or tumors [40]. To optimize the efficiency of Ag presentation by DCs requires that they encounter CpG ODN in the presence of vaccine Ag. Co-delivery of ODN plus Ag to the same APC accelerates the induction, increases the maximal level and extends the duration of the induced immune response [41]. It also supports modulation of Ab isotype and increases the immunogenicity of weak Ags [42]. Examples include studies in which ovalbumin or the hepatitis B surface antigen vaccine were administered with CpG ODN in which co-delivery to the same site significantly enhanced humoral protective immunity [21,43,44]. 

Based on such findings, a number of delivery strategies were examined to optimize the co-delivery of CpG ODN plus Ag to the same APCs. These approaches included the preparation of CpG-Ag conjugates, co-encapsulation in liposomes or on biodegradable microparticles, and the use of multicomponent nanorods [40,45,46]. Murine studies show that conjugating CpG ODN directly to Ag can boost immunity by up to 100-fold over that induced by simply mixing CpG ODN with immunogen [47,48]. The mechanisms by which CpG ODN-Ag conjugates enhance immunogenicity include insuring that both Ag and TLR agonist are taken up by the same APC and improving such uptake via DNA-binding receptors on the APCs (the latter effect is independent of the nature of the ODN but requires physical conjugation of DNA to target antigen). 

While early murine studies focused on administering CpG ODN with defined vaccine Ags, their ability to support immunity when combined with complex vaccines expressing multiple tumor Ags has also been examined. One such endeavor examined the effect of conjugating CpG ODN to apoptotic tumor cells [49]. Whole mouse tumor cells were used because they expressed all possible tumor-associated Ags, allowing the host’s immune system to select the most immunogenic determinant based on presentation in the context of self MHC. Apoptotic tumor cells alone lack a TLR signaling moiety and thus fail to trigger the innate immune system in support of tumor specific immunity. To overcome this limitation, CpG was conjugated directly to tumor cells. The resultant adjuvant/vaccine combination triggered the expansion of tumor-specific CTL in the periphery that reduced the growth of small tumors and prevented their metastatic spread in murine experiments [49].

There is concern that inclusion of CpG ODN may increase the risk of vaccine-induced autoimmunity. CpG ODN and immune complexes that contain nucleic acids interact with TLR9 to increase the production of type I IFNs. While IFNa and IFNb can induce/exacerbate autoimmune disease [50,51,52,53], whether CpG based adjuvants have such an effect remains controversial, having not been observed in clinical vaccine trials [40,54]. We conclude that when a vaccine against cancer capable of overcoming tolerance to tumor Ags is required, the benefit of including a strong adjuvant (such as CpG ODN) outweighs the potential risk.

## 6. Effect of CpG DNA on MDSC and Macrophages in the Tumor Microenvironment

The anti-tumor activity of CpG-adjuvanted tumor vaccines was initially examined by delivering the vaccines systemically (by i.m. or s.c. routes) [55]. In murine studies, CpG-adjuvanted vaccines were effective against small tumors (<300 mm^3^) but were unable to eliminate large established tumors (of the size typical present in humans when first diagnosed). Whether delivered to mice with large or small tumors, the CpG-adjuvanted vaccines continued to induce tumor-specific CTL that were readily detected in the peripheral circulation. The problem was that immunosuppressive leukocytes present in the microenvironment of large tumors down-regulated the activity of these CTL. To overcome this limitation, CpG ODN were injected directly into the tumor bed with the goal of activating intratumoral DCs and facilitating tumor Ag presentation *in situ*.

Unexpectedly, local delivery interfered with the function of tolerogenic cells in the tumor milieu. Intra-tumoral injection of CpG ODN reduced the number and suppressive activity of tumor infiltrating monocyte-derived suppressor cells (MDSC) [56]. This was true of both free and vaccine associated CpG ODN, and led to a re-interpretation of data from clinical trials in which CpG ODN were delivered intratumorally to treat skin tumors or lymphoma. For example, Hofmann *et al.* induced complete or partial tumor remission in half of all patients with basal cell carcinoma or melanoma by intra-tumoral CpG injection [57]. Molenkamp *et al.* showed that intratumoral CpG increased the frequency of tumor-specific CD8 T cells in half of patients with melanoma [58]. Brody *et al.* showed that intratumoral CpG administration combined with radiation therapy induced systemic tumor regression (including at untreated sites) and tumor-reactive CD8 T cells in patients with low-grade B-cell lymphoma [59]. Kim *et al.* showed intratumoral injection of CpG ODN combined with radiation induced the regression of distal tumors, significantly decreased the frequency of FoxP3+ regulatory T cells (Tregs) and increased the frequency of CD123+ pDC at the site of CpG administration in patients with lymphoma [60]. These strategies are referred to as “*in situ* tumor vaccination” as they do not require the use of a customized vaccine. 

These findings are consistent with animal studies showing that local CpG ODN treatment increased the number of tumor infiltrating T and NK cells while decreasing the frequency and inhibitory activity of tumor resident MDSC. The monocytic MDSC studied in that work expressed TLR9 and exposure to CpG ODN (i) triggered their rapid production of Th1-type cytokines (including IL-6, IL-12 and TNFa); (ii) impaired their ability to secrete arginase 1 and nitric oxide (factors critical to their suppression of T cell activity) and (iii) induced them to differentiation into tumoricidal macrophages [56]. These results suggest the existence of additional mechanisms through which CpG ODN could promote tumor regression. Unfortunately, human mMDSC do not express TLR9 or respond to CpG ODN, limiting the clinical applicability of the murine findings. However, we find that the suppressive activity of mMDSC isolated from cancer patients can be reversed by treatment with TLR 7/8 agonists which induce them to differentiate into tumoricidal M1-like macrophages in a manner very similar to CpG ODN in mice [61].

## 7. TLR 7 and TLR8 Agonists as Cancer Vaccine Adjuvants

TLR7 and TLR8 are closely related receptors. They have similar structures and trigger a similar signaling cascade but differ in their pattern of cellular expression and thus the array of cytokines they elicit. In humans, TLR7 receptors are present on B cells and pDC which when activated secrete IFNa. TLR8 receptors are prevalent on neutrophils, monocytes and mDC which when triggered secrete TNFa, IL-12 and MIP1a [62,63]. Most ligands that interact with TLR7 also bind to TLR8. These include synthetic imidzaquinolines (such as resiquimod/R-848), and the natural ligand ssRNA containing GU-rich sequences. Imiquimod and the guanosine analogue loxoribine are considered selective for TLR7 [64,65,66]) and a new generation of TLR8 selective agents has been described [63].

## 8. Trials Utilizing TLR 7/8 Agonists

The utility of TLR 7/8 ligands as vaccine adjuvants was evaluated in pre-clinical studies. Smorlesi *et al.* used transgenic mice expressing the HER2/neu oncogene that spontaneously develop mammary tumors [67]. When immunized with a DNA vaccine plus imiquimod, the incidence and growth rate of breast tumors was reduced when compared to DNA vaccination alone. Ab titers in mice receiving the imiquimod adjuvanted vaccine were higher and biased towards IgG2a and the number of CD8 T cells producing IFNg was also increased [68]. Narusawa *et al.* evaluated imiquimod as an adjuvant when used in combination with GM-CSF plus a gene-transduced tumor vaccine (GVAX). This vaccine combination significantly reduced the rate of tumor growth while increasing the number of pDC [69]. It should be noted that neither of these studies examined the effect of TLR 7/8 adjuvanted vaccines on large established tumors, limiting the ability to draw conclusions concerning their utility under conditions similar to those found in patients with cancer. 

The TLR7/8 agonists currently approved by the FDA are designed for topical administration and are used primarily to treat HPV-induced warts, lentigo maligna, actinic keratoses, and basal or squamous cell carcinoma [70,71,72,73]. Clinical studies therefore routinely relied on topical administration to evaluate TLR 7/8 ligand activity. In a trial of patients with prostate cancer characterized by rising PSA titers (indicative of tumor growth), a prostate-specific peptide vaccine was combined with one of several adjuvants or immunomodulatory treatments. Patients receiving topical imiquimod over the vaccine injection site had the best clinical outcome with the slowest rise in PSA when compared to other modalities (including GM-CSF, hyperthermia, and mucin-1-mRNA/protamine complex) [74].

The ability of imiquimod to act as a topical adjuvant was also evaluated in patients with melanoma. When administered in conjunction with a variety of melanoma specific peptides plus Flt3 ligand, imiquimod stimulated an increase in the frequency of peptide-specific CD8 T cells [75]. When used in combination with a vaccine containing the NY-ESO-1 cancer Ag, four out of nine patients with melanoma developed specific Abs while seven out of nine developed CD4 T cell responses. CD8 T cell responses were not enhanced in these subjects nor did disease progression correlate with the induction of the types of immunity observed [76]. In an effort to improve outcome, resiquimod was substituted for imiquimod based on animal studies showing that this TLR 7/8 agonist was better at generating Ag specific CD8 T cells [77,78]. A clinical study of NY-ESO-1 immunized melanoma patients found that the addition of resiquimod improved CD8 T cell responses in 25% of patients. No change in Ab or CD4 T cells was observed nor was time to progression improved (interestingly CD8 T cell responders also expressed the TLR7 SNP rs179008) [79].

## 9. Trials Utilizing CpG ODN

Agonists targeting TLR9 have been studied more extensively than those against TLR7/8. CpG ODN showed activity in murine models as monotherapy, in combination with cancer vaccines, and when paired with other modalities including radiotherapy, cryotherapy and chemotherapy. Numerous preclinical studies showed that including CpG ODN increased CTL frequency and that this effect correlated with slower tumor progression. For example, the immunogenicity of DC-based tumor vaccines was improved by the addition of CpG ODN as characterized by a marked improvement in CD8 T cell activity [80]. Most such studies delivered the CpG adjuvanted vaccines before or shortly after challenge and thus targeted tumors that were relatively small [81,82,83,84,85,86]. However, recent studies indicate that CpG ODN adjuvanted vaccines can eradicate even large established tumors. In one report, combining CpG ODN with a peptide vaccine targeting HPV16 E7 resulted in the elimination of tumors up to 250 mm^3^ in size. The growth of even larger tumors was significantly delayed although eradication was not achieved [87]. Eradication of very large tumors (1.2 cm in diameter) was observed in 50% of mice using a fusion protein vaccine targeting the E7 epitope in combination with CpG ODN plus a chemotherapeutic agent [88].

Several clinical trials examined the use of CpG ODN combined with peptide-based vaccines targeting tumor antigens. These studies commonly evaluated additional immunostimulatory agents such as Montanide ISA-51, GM-CSF and IFA. Phase I trials of the MART-1 peptide vaccine in patients with melanoma reported that the inclusion of CpG ODN increased the number of Ag-specific CD8 T cells by 10-fold [89,90]. Higher levels of IFNg, TNFa, and IL-2 were also detected [91]. In another trial using a multi-epitope peptide vaccine that included MART-1, gp100, and tyrosinase 40%–50% of patients developed IFNg secreting CD8 T cells and two-thirds of these had stable disease or partial regression. Unfortunately, these benefits lasted only 2–7 months and did not result in a significant difference in outcome when compared to other therapies in patients with stage IV or recurrent melanoma [92]. Expansion of CD8 T cells was also observed in studies utilizing the NY-ESO-1 peptide. In two trials, three out of three and nine out of 18 patients responded [93,94]. Elevated CD8 T cell responses were also observed against cancers expressing the NY-ESO-1 or LAGE-1 tumor Ag, and such responses were associated with improved clinical outcomes [95].

CpG ODN were also evaluated in combination with a vaccine targeting the Wilms’ Tumor-1 Ag (WT-1). Among patients receiving the CpG adjuvanted vaccine, 60% had stable disease compared to 15%–20% of those lacking the CpG component [96]. A study of patients with metastatic esophageal squamous cell carcinoma used a vaccine targeting the cancer-testis Ag peptides LY6K and TTK. Inclusion of CpG ODN led to an increase in CD8 T cells and secretion of IFNa. More patients receiving the CpG vaccine had stable disease compared to patients who did not (33% *vs.* 66%) although no complete or partial remissions were induced [97].

A reasonable conclusion from these human trials is that the addition of TLR adjuvants modestly boosts vaccine induced immunity but rarely results in tumor eradication. One explanation for this limited success is the ability of established tumors to evade immune elimination. The microenvironment in which tumors reside is rich in factors that support growth and contains Tregs and MDSCs that down-regulate tumor-specific immunity [98,99]. Tregs aid the host by suppressing autoreactive T cells and thus prevent autoimmunity [100,101,102]. However, when present in the tumor milieu they disrupt the host’s ability to destroy cancer cells [100,103,104]. Many tumors actively secrete factors such as CCL2 that recruit Tregs or that induce naive T cells to differentiate into Tregs [105,106,107]. Myeloid-derived suppressor cells are also present at high frequency in established tumors. They inhibit the tumoricidal activity of T and NK cells by interfering with l-arginine metabolism through the production of Arg-1 and iNOS or ROS [108,109]. Tregs and MDSC within the tumor microenvironment thus block the effector function of immune cells generated by TLR adjuvanted vaccines [110,111].

One way to limit the activity of these immunosuppressive cells is to induce their differentiation. For example, TGFb, IL-10 and other factors can induce Tregs to differentiate while IL-6, IL-10 and TNFa can drive MDSC to differentiate into macrophages [100,112,113,114]. Intratumoral delivery of CpG ODN has been shown to slow tumor growth by altering the balance between suppression and immunity. While TLR9 stimulation increases systemic production of NK and CD8 T cells [55,115,116,117], local delivery improves tumor infiltration by such cells. Moreover, murine studies show that local delivery reduces the frequency of immunosuppressive Tregs and monocytic MDSCs in the tumor microenvironment [55,115,116,117]. *In vitro* studies demonstrate that MDSCs lose their immunosuppressive activity when treated with CpG DNA in association with reduced expression of NO and Arg-1 [56,118,119]. These effects were driven by the differentiation of mMDSC into tumoricidal M1 macrophages (as characterized by decreased expression of Ly6c and Gr-1 and increased expression of F4/80). Transferring these differentiated cells into tumor-bearing animals significantly slowed tumor growth, indicating that intra-tumoral delivery of CpG ODN (alone or in conjunction with vaccine) might profoundly alter the balance between tumoricidal and immunosuppressive cells [118,119].

Recent reports suggest that TLR 7/8 agonists also trigger MDSC maturation. Resquimod induces murine MDSC to differentiate into F4/80+ macrophages and CD11c+/I-Ad+ dendritic cells that support the expansion of CD4 and CD8 T cells [120]. Our group found that mMDSCs isolated from the peripheral blood of normal volunteers and cancer patients differentiated into M1-like macrophages when exposed to several TLR7 and TLR8 agonists. Interestingly, this was not a universal effect of all TLR agonists. PAM3, a ligand for TLR1/2, causes mMDSC to differentiate into M2-like macrophages that support tumor growth [61]. TLR7 agonists have additional effects on immune cells. For example, loxorubin can inhibit tumor growth by promoting CD4 T cell proliferation and modulating the suppressive activity of Tregs [121]. 

## 10. TLR Agonist Combinations

Many strategies have been identified that enable the immune system to eliminate small tumors in animal models (including the intra-tumoral delivery of TLR agonists). Such strategies become increasingly less effective as larger cancers are targeted, in part because large tumors are infiltrated by immunosuppressive cells that inhibit the activity of tumoricidal CLT and NK cells [113,122,123]. Our group examined the effect of combining a novel TLR7/8 agonist (3M-052) with CpG ODN. While each TLR agonist alone slowed tumor growth, neither prevented the eventual outgrowth of established CT26 cancers. In contrast, the combination of both agonists cleared large established tumors in 87% of mice [119]. Mechanistically, this combination of TLR agonists reduced the number of mMDSCs and increased the number of CD8 T cells much more effectively than either agent alone. Intra-tumoral delivery of this TLR agonist combination up-regulated the expression of IL12, IFNg and granzyme B while lowering levels of Arg-1, Nos 2, CTLA-4 and TGFb [119]. The effect of combining TLR7 plus TLR9 agonists was evaluated in a single clinical trial. A virus-like nanoparticle containing CpG ODN plus the melanoma protein MelQbG10 was used in conjunction with imiquimod. The MelQbG10/CpG vaccine elicited tumor specific CD8 T cell responses. Inclusion of the TLR7 agonists significantly increased the magnitude of that response and improved the generation of memory T cells [124].

TLRs 7, 8 and 9 are all endosomal receptors, are expressed on overlapping cell types, and utilize similar signaling pathways [62,63,125]. What then accounts for their synergistic anti-tumor activity (particularly since TLR7 triggering may inhibit cytokine secretion induced by TLR9 agonists) [126,127]? A recent report shows that the expression of receptors by individual cells is stochastic and that cells with high levels of one receptor can have much lower levels of another [128]. We postulate that a greater fraction of APCs are activated by the combination of TLR7 plus TLR9 agonists than by either alone. Consistent with such a conclusion, the fraction of MDSC activated to differentiate and secrete cytokines was significantly increased when these cells were stimulated with both agonists *vs.* either one singly [119].

First generation TLR7/8 agonists were short acting agents designed for topical use. A new generation designed for *in vivo* administration and use as vaccine adjuvants is now available. Preliminary studies indicate that they are safe and persist at the injection site [63]. Delivering these agents in combination with CpG ODN into the tumor microenvironment as adjuvants for tumor vaccines thus represents a promising approach to the immunotherapy of large established cancers.

## 11. Conclusions

TLR agonists have complex and pleiotropic effects on the immune system. When used as adjuvants, TLR agonists boost Ag-specific cellular and humoral immunity. Stimulating endosomal TLRs is particularly effective at promoting the generation of CTL capable of eliminating viral pathogens and cancer. Simultaneous activation of multiple TLRs further improves the breadth and efficacy of such responses. When targeting cancer, intra-tumoral delivery of agonists against TLRs 7, 8 and 9 provides the added benefit of altering the tumor microenvironment. Such treatment reduces the frequency of immunosuppressive Tregs, MDSC and M2 macrophages while increasing the frequency of tumoricidal M1 macrophage. We believe that intra-tumoral delivery of vaccines that include TLR agonists should be of considerable benefit in the immunotherapy of cancer.
